# Cytokine “fine tuning” of enthesis tissue homeostasis as a pointer to spondyloarthritis pathogenesis with a focus on relevant TNF and IL-17 targeted therapies

**DOI:** 10.1007/s00281-021-00836-1

**Published:** 2021-02-05

**Authors:** Tobias Russell, Charlie Bridgewood, Hannah Rowe, Ala Altaie, Elena Jones, Dennis McGonagle

**Affiliations:** 1 Leeds Institute of Rheumatic and Musculoskeletal Medicine (LIRMM), University of Leeds, Leeds, UK; 2 Section of Musculoskeletal Disease, Leeds Institute of Molecular Medicine, University of Leeds, NIHR Leeds Musculoskeletal Biomedical Research Unit, Chapel Allerton Hospital, Leeds, UK

**Keywords:** Spondyloarthritis, Enthesis, IL-17, TNF

## Abstract

A curious feature of axial disease in ankylosing spondylitis (AS) and related non-radiographic axial spondyloarthropathy (nrAxSpA) is that spinal inflammation may ultimately be associated with excessive entheseal tissue repair with new bone formation. Other SpA associated target tissues including the gut and the skin have well established paradigms on how local tissue immune responses and proven disease relevant cytokines including TNF and the IL-23/17 axis contribute to tissue repair. Normal skeletal homeostasis including the highly mechanically stressed entheseal sites is subject to tissue microdamage, micro-inflammation and ultimately repair. Like the skin and gut, healthy enthesis has resident immune cells including ILCs, γδ T cells, conventional CD4+ and CD8+ T cells and myeloid lineage cells capable of cytokine induction involving prostaglandins, growth factors and cytokines including TNF and IL-17 that regulate these responses. We discuss how human genetic studies, animal models and translational human immunology around TNF and IL-17 suggest a largely redundant role for these pathways in physiological tissue repair and homeostasis. However, disease associated immune system overactivity of these cytokines with loss of tissue repair “fine tuning” is eventually associated with exuberant tissue repair responses in AS. Conversely, excessive biomechanical stress at spinal enthesis or peripheral enthesis with mechanically related or degenerative conditions is associated with a normal immune system attempts at cytokine fine tuning, but in this setting, it is commensurate to sustained abnormal biomechanical stressing. Unlike SpA, where restoration of aberrant and excessive cytokine “fine tuning” is efficacious, antagonism of these pathways in biomechanically related disease may be of limited or even no value.

## Introduction

**Fig. 1:**
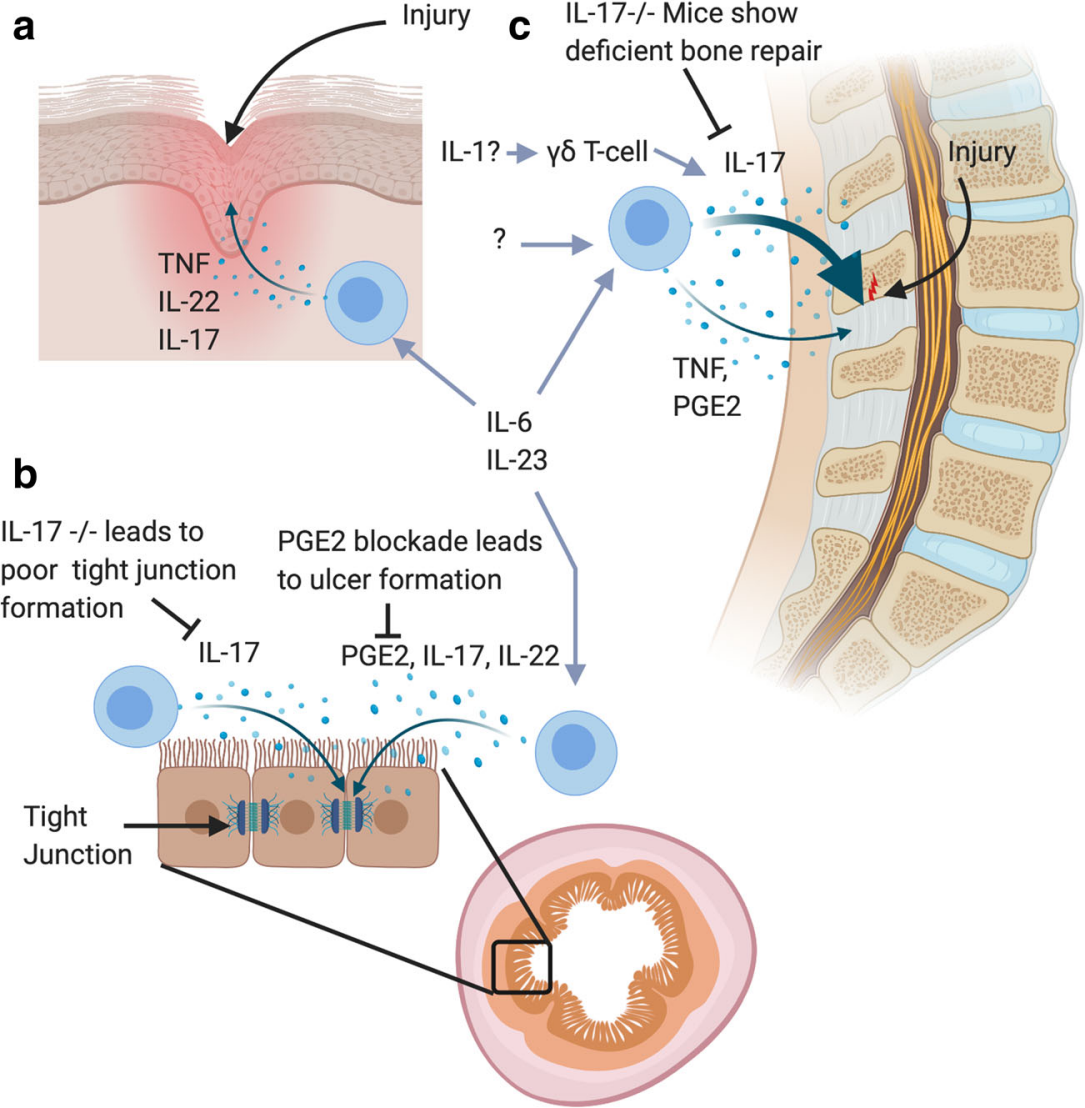
The spondyloarthropathy target tissues beyond the spine including the gut and the skin have a well-established understanding whereby the immune system plays a key role in physiological tissue repair. **a** Damage to the skin leads to rapid influx of immune cells producing cytokines including TNF, IL-17 and IL-22 which are involved in keratinocyte proliferation and extra cellular matrix deposition. **b** The evidence for microdamage to the spinal enthesis and how this regulates of IL-17A, TNF and PGE2 at entheses and tendons is discussed in the text. Mouse knockouts for IL-17A show impaired fracture healing responses which is rescued by IL-17A supplementation. **c** The hostile enzymatic and microbial normal gut environment is associated with tissue microdamage. IL-23, IL-22, IL-17A, IL-22 or PGE2 play key roles in gut homeostasis in experimental models. In particular, IL-17A plays a pivotal role in the tight junction formation between cells are damaged resulting in leaky gut

The seronegative spondyloarthropathies (SpA) include ankylosing spondylitis (AS), non-radiographic axial SpA (nrAxSpA) are associated with post-inflammatory excessive tissue repair responses manifesting as new bone formation [1]. This is in contra-distinction to rheumatoid arthritis (RA) where inflammation is associated with predictable bone and cartilage destruction [2]. Beyond the skeleton, including the axial skeleton, the skin, gut and occasionally the arterial tree are target tissues in the axial SpA. The skin, gut and aortic root are biomechanically stressed or chemically stressed sites that are associated with damage and repair responses [1, 3]. There is a considerable body of literature describing how different immune cells and cytokines including TNF, IL-23, IL-22, IL-17 family member cytokines and others are involved in tissue repair responses [4–6] (Fig. 1). The role of these molecules in the skin and gut homeostasis is not the subject of this perspective and is well reviewed elsewhere [7, 8]. The purpose of this article is to apply this concept to understanding axial SpA in the context of entheseal biology and translationally relevant cytokines and other molecules and to consider the same concept in relationship to degenerative enthesopathy and tendinopathy.

Therapeutically, AS responds to anti-cytokine targeting including anti-TNF, anti-IL-17 blockers and also good responses to cyclooxygenase (COX) enzyme inhibition [9–11]. In SpA, there is evidence for peripheral PsA responding to anti-IL12/23 or anti-p19 IL-23 blockers [12] but lack of evidence for efficacy in axial disease in AS [13] that suggests a key role for IL-17A biology in the spine that requires independent functioning of IL-17A from IL-23. The non-steroidal anti-inflammatory drugs (NSAIDs) are remarkably effective for the therapy of human AS with many patients demonstrating good and sustained responses with inflammation and sub-fibrocartilage neo-vascularisation abrogation as shown on imaging [14]. That collectively in some of these pathways (COX and IL-17 axis) inhibition is so effective in AS but not RA empirically shows that these are central to the pathology of axial SpA. Herein, we describe how excessive activity in these translationally relevant pathways may have pivotal roles in repair of SpA-related target tissue. Data from enthesis cellular immunology and translational human immunology are synthesized to explain how cytokine “fine tuning” of enthesis homeostasis and repair could be a key consideration for understanding axial SpA spectrum disease. The corollary to this is that cytokine production in “non-inflammatory” or biomechanically induced or degenerative enthesopathy may also have a role in cytokine “fine tuning” of repair to restore homeostasis. Such a scenario is not without support given that the pan-cytokine inhibition of corticosteroids may actually lead to Achilles tendon rupture [15].

## Linking immunity and spinal tissue repair—immunity

The enthesis is under constant mechanical stress and in SpA relevant disease models this results in a pro-inflammatory cytokine production and inflammatory cell recruitment in a biomechanically stress dependent mechanism [16–18]. In humans, the post-inflammatory phase of axial enthesitis is associated with complete spinal fusion and a “bamboo spine” [19]. In the normal human enthesis, several immune cell types have been identified including, type 3 ILC’s (innate lymphoid cells) [20], natural killer (NK) cells [21], γδ T cells [22] and conventional αβ CD4+ and CD8+ T cells [23]. This emergent enthesis immune system is reminiscent of that in both the skin and the gut [24], but the absence of local microbiotal communities at the enthesis raises important issues as to why such an immune system is compartmentalized at the enthesis.

GWAS studies in AS have implicated specific immune pathways associated with pathogenesis, including the IL-17/IL-23 pathway, control of NF-κB activation, amino acid trimming via ERAP-1 for MHC antigen presentation and other genes controlling CD4+ and CD8+ T cell subsets [25]. The GWAS data incriminating lymphocytes in axial SpA is supported by rudimentarily immunology where both innate and conventional αβ T cells found at the normal human enthesis have inducible key SpA associated cytokines, IL-17A and TNFα [22, 23, 26]. In the context of experimental SpA pathogenesis, excessive TNF or IL-17 driven immune systems activation are associated with a primary enthesitis, which may later spread to the adjacent tissues [16–18]. Of course, the TNF and IL-17 pathways have been successfully targeted in humans providing incontrovertible proof of a pivotal disease role [27, 28].

## Linking immunity and spinal tissue repair—tissue repair and new bone

Important to tissue repair are resident mesenchymal stem cells (MSCs), which have been identified in almost every tissue in the human body. MSCs display a receptiveness towards an array of chemokines and cytokines which are all affiliated with tissue repair following on from injury or damage [29–31]. Notably, cytokines including IL-17A are elevated at fracture sites and capable of driving osteogenesis from local MSC populations [32, 33]. What is also recognized in AS that NSAIDs are generally associated with the suppression of new bone formation [34] with the exception of one study which is limited by the fact that the control arm could take NSAIDs on demand [35]. In the orthopaedic setting, there is a general aversion of NSAID use for analgesia in the setting of post-operative spinal fusion surgery due to the negative impact on bone union of surgically desired segmental fusion [36, 37]. Indeed, several studies show that NSAIDs hinder mesenchymal stem cell osteogenesis [38]. Rheumatologists may not be aware but a strong interest in developing prostaglandin (PGE)2 agonists for their known bone building capabilities was mooted more than 20 years ago [39].

Whilst the role of COX inhibition on skeletal biology is fairly well defined, the role of the pivotal therapeutically antagonized cytokine molecules is less well defined. A highly pertinent observation is that the pivotal cells involved in the osteogenesis in AS, namely MSCs and other stromal cells express both TNF-R1 and IL-17RA, in addition to COX enzymes so are potentially directly impacted by licenced therapy for AS [31, 40]. Having defined the entheseal cellular players, we look at molecules and cytokines known to be pivotal in axial enthesitis in AS in the context of tissue repair fine tuning.

## Prostaglandin E2

Prostaglandin E2 (PGE2) can be produced by almost every cell in the human body, it is a lipid mediator that is synthesized from arachidonic acid via COX enzymes [41]. Prostaglandin E2 exerts its effects through four different G protein-coupled receptors (EP1-EP4). EP4 is of particular interest for potential roles in AS [42] and normal bone formation [43]. Prostaglandin E2 has long been known to cause an increase in osteogenesis, with immobilized femurs of female rats treated with PGE2 showing increased osteogenesis in these immobilized bones [44]. Of considerable interest after a GWAS in AS was a single nucleotide polymorphism (SNP) at chromosome 5p13 [42]. The rs10440635 SNP in the prostaglandin E receptor 4 (PTGER4) gene is robustly associated with AS [42]. PTGER4 encodes the prostaglandin E2 receptor 4 (EP4), which when activated is seen to amplify CD40 mediated induction of IL-23 p19 expression [45]. The evidence that NSAIDS are highly effective at reducing new bone formation linked to PGE2 expression suggests at a potential role [14]. However, 2-year continuous treatment using the NSAID diclofenac did not significantly reduce radiographic progression in AS patients compared to an on-demand treatment of the same NSAID [35], which is in contrast to similar studies involving celecoxib which showed that continuous usage over a 2-year period did decrease radiographic progression of AS when compared to an on-demand treatment [46]. The Wanders et al. study is however limited in its findings; notably only 65% of patients completed the study without switching to another NSAID with their control arms also taking NSAIDs when undergoing flares which likely trigger the cycle of inflammation [46]. With NSAIDs all showing comparable levels of COX-2 inhibitions [47], the use of COX-2 selective and non-COX selective inhibitors does have an impact on bone formation. With celecoxib showing no clear difference in the prevention of hip ossification after replacement when compared to indomethacin [48], whilst another study showed superior inhibition of hip ossification for the same surgery when compared to ibuprofen [49].

More recently, co-culture models of classically activated M1 macrophages and MSCs demonstrated enhanced mineralisation associated with PGE2 secretion by the M1 macrophages in early phases of MSC differentiation, compared to MSCs cultured alone [39]. A significant decrease in mineralisation observed with COX-2 inhibitor was added to the culture [39]. With macrophages being elevated early at fracture sites [50], this suggests at a role for PGE2 in normal fracture repair. Murine COX-2 knockouts showed a delayed initiation and an impaired endochondral bone repair that was associated with severe angiogenesis impairment [51]. Agonists for EP4 or EP2 can rescue impaired fracture healing seen in COX-2 knockout mice [51]; they can also augment normal fracture repair in rat fracture models [52].

## Tumour necrosis factor

TNF exerts its affects through two different receptors: TNFRI/p55 and TNFRII/p75 [53]. TNFRI is expressed ubiquitously and constitutively, whilst TNFRII is only expressed on immune cells. TNF is mainly produced by myeloid cells such as macrophages, but other immune and stromal cells are capable of secretion [40]. TNF has an established role in healthy fracture repair, with TNF receptor knockouts of both TNFRI and TNFRII demonstrating delay MSC migration and a subsequent delay in endochondral tissue formation [30]. Supplementation using TNF in fracture sites results in accelerated bone repair with greater callus mineralisation 28-days post fracture [54, 55]. The migration of MSCs towards fracture sites is also seen in humans with significantly elevated levels of TNF after 72-h seen in the peri-fracture area [56], which helps drive MSC migration by upregulating expression of intercellular adhesion molecule (ICAM)-1 and vascular adhesion molecule (VCAM)-1 [57]. However, it is important that levels of TNF at the fracture site are controlled with prolonged elevated levels working synergistically with IL-1β resulting in increased chondrocyte apoptosis and impaired chondrocyte proliferation [58]. This is associated with a decrease in subsequent new bone formation at the callus site. Interestingly, TNF stimulates tissue nonspecific alkaline phosphatase and subsequent mineralisation in the presence of collagen, independent of the RUNX2 pathway [59]. With the collagen rich environment at the enthesis, this ectopic mineralisation of the collagen fibrils could be acting as a template for calcium crystal deposition and potentially assisting in the new bone formation at the enthesis.

Elevated levels of TNF are seen to induce persistent elevation of Wnt proteins. Elevation activates NF-κB (p65) and c-Jun N-terminal kinase (JNK)/activator protein-1 signalling pathway leading to bone formation. However, both the canonical and non-canonical pathways are needed to induce bone formation via inflammation with inhibition of either pathway significantly decreasing bone formation [60]. Though TNF is shown to elevate levels of Dickkopf-1 (DKK-1), this is shown in mouse models to suppress Wnt signalling, allowing for enhanced bone resorption with an increase in osteoclast activator RANKL [61]. Inhibition of DKK-1 resulted in increased bone formation via Wnt signalling activation, with subsequent RANKL mediated bone resorption being blocked by osteoprotegerin (OPG) [61].

In AS, the role that DKK-1 remains contentious with various studies and meta-analyses showing contrasting results with some showing significant elevation of DKK-1 in AS [62, 63], others showing a significant decrease [64, 65] whilst some showed no significant differences [66, 67]. There is some evidence DKK-1 is dysfunctional in AS and a result is capable of abnormal activation of β-catenin independent Wnt signalling by binding less avidly to low-density lipoprotein receptor-related protein 6 (LRP6), a key receptor in the canonical Wnt pathway [68]. With the mixed literature on its potential affects it remains unclear what role it plays in the pathogenesis of AS. Whilst it appears that TNF can indeed fine tune or increase osteogenesis, the idea that new bone formation occurs in AS via DKK-1 in face of high TNF needs further evaluation.

## IL-17A and other IL-17 family cytokines in tissue repair

IL-17A elevation was shown to contribute to fracture repair shortly after injury with γδ T cells being the primary source [32, 33]. The reasoning for this increased osteogenesis is mixed within the literature with IL-17A shown to both increase osteogenesis [32] but in rat calvarial cells to decrease osteogenesis [69]. The difference in effects IL-17A has on osteogenesis is suggested to be dependent on the target cells origin. Immature mesenchymal cells such as MSCs are reported to display increase osteogenesis with exposure to IL-17A [31, 70], whilst calvarial pre-osteoblasts are seen to show decreased osteogenesis [69, 71].

Generally, it is thought that IL-17A elevation in the aftermath of a bone injury promotes bone regeneration, with murine IL-17A−/− models displaying impaired bone regeneration following drill hole in the femur at 14 and 21 days post injury compared to wild-type mice [32]. Elevation of IL-17A activates osteoblasts via JAK2/ signal transducer and activator of transcription (STAT) 3 signalling pathway, causing osteogenesis and fracture repair [31]. Inhibition of IL-17A in psoriatic arthritis (PsA) trials showed substantial reductions in small joint erosion, associated with inflammation suppression [72, 73]. As with PsA, AS shows similar elevated levels of IL-17A in both the serum and synovial fluid of patients [31, 74].

Emerging evidence suggests an important role for IL-17F in SpA spectrum disease and psoriasis since dual suppression of IL-17A and IL-17 may offer benefit, especially in the skin [75]. IL-17F expression following inflammatory stimuli can activate the CCAAT/enhancer binding protein (C/EBP)-β that mediates osteoblastogenesis seen in early fracture repair [76]. This is supported by a mouse tibial fracture model where IL-17F is seen to be elevated 3 days after the fracture at the fracture callus demonstrating a role in fracture repair [77]. More emerging evidence is showing a synergy between IL-17A and IL-17F where dual inhibition of both cytokines has a greater osteogenic suppression than inhibition of either one cytokine alone, with stimulation of periosteum-derived cells by either IL-17A or IL-17F showing equal levels of increase of osteogenesis [78].

Rag1 knocked-out mice, which prevent development of mature B and T cells, demonstrated impaired fracture healing compared to wild types and interestingly IL-17F was shown to rescue this impaired osteogenesis [77]. This increased osteogenesis by IL-17F has not only been reported on MC3T3-E1 cell lines [76], but it also increased human osteoblastic differentiation from human and mouse bone marrow derived MSCs [70, 77]. Clearly from these experiments, it will be important to ascertain whether IL-17F dysregulation could play a role in AS related inflammation and post inflammation tissue remodelling. Of particular interest was the demonstration of two IL-17F gene polymorphisms (7383A/G and 7488A/G) associated with AS disease in a Turkish patient cohort [79].

The biology of other IL-17 family members is incompletely understood and given the role of IL-17A/F these are briefly summarized. Elevated levels of IL-17RB are seen in the first 2 weeks post fracture in rat long bones, with it localized to trabecular bone osteoblasts, primitive and pre-hypertrophic chondrocytes as well as localized MSCs [80]. The exact role it might play is not known or well-studied. The compelling evidence that these cytokines are involved in SpA like diseases from murine collagen-induced arthritis (CIA) models. The CIA model shows a prominent inflammatory entheseal component, which was previously regarded as an experimental bulwark of RA pathogenesis [18]. With both IL-17C and IL-17B mRNA being elevated in the arthritic paws of CIA mice, the disease is ameliorated with the addition of IL-17B antibodies [81]. Moreover, IL-17C enhanced the production of TNF from murine peritoneal exudate cells, with IL-17C-transduced CD4+ T cells exacerbating the arthritic destruction [81]. Interestingly, the addition of IL-17E to a CIA mouse model attenuated the disease state [82]. This is associated with IL-17E role in inhibiting the differentiation of Th17 cells from CD4+ T cells, causing a subsequent reduction in pro-inflammatory cytokines associated with arthritis [82, 83]. Recent work on SpA synovial tissues has found that IL-17D is the most expressed IL-17 family member, with downregulation in SpA-simulated inflammation suggesting a potential anti-inflammatory role in SpA though further work is needed to fully establish its role [84].

## Cytokines that indirectly impact on the TNF or IL-17 pathways at the enthesis

**Fig. 2:**
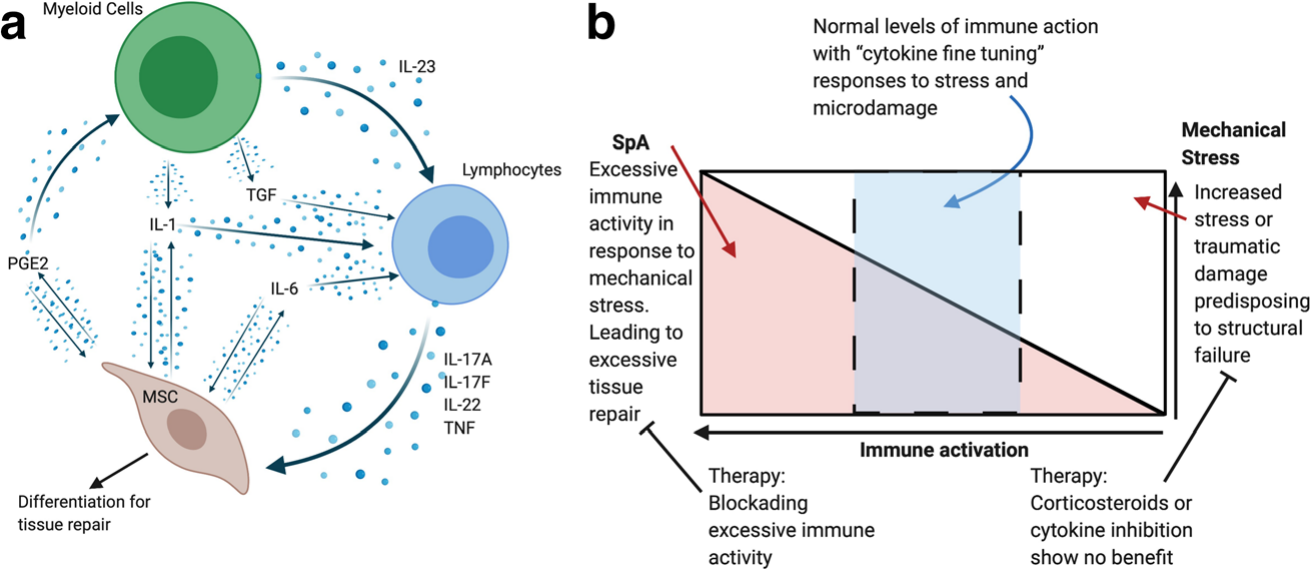
**a** Beyond TNF and IL-17A other cytokines are indirectly involved in the axis. For example, IL-1, IL-6 and TGF-beta can prime IL-17A, IL-17F and IL-22 production and genetic polymorphisms in IL-1 an IL-6 are linked to AS. Independently of the impact on IL-17 pathway biology these cytokines can also directly impact on stromal and MSC biology. **b** The balance between immune activation in response to mechanical stress. Overactivation of the immune system in response to mechanical damage can lead to excessive repair as seen in SpA (red). Conversely blocking the immune system when excessive mechanical stress induced injury can be detrimental to the repair process or may render repair ineffective

Many cytokines regulate IL-17 axis cytokines or are the product of IL-23/17 axis stimulation and include IL-1, IL-6, TGF-β and others and these are briefly discussed (Fig. 2). The IL-1 pathway has repeatedly been identified in GWAS in AS [85, 86]. It is now known that IL-17 production can occur independently of IL-23 stimulation [87] and that IL-1β is capable of driving maturation of IL-17 producing cells [88, 89]. This supports a role for the IL-1 family in AS especially since IL-23 blockade is ineffective for spinal disease progression [90, 91]. IL-1β is capable of inducing the expression of other inflammatory cytokines from CD4+ and CD8+ cells; this allows for an initiation of an inflammatory response seen in tissue repair. IL-1β along with IL-23 is capable of inducing the expression of IL-17 from Th17 cells, but IL-1β is also shown to induce the expression of IL-17, IL-21 and IL-22 from γδ T cells due to their expression of the transcription factor RORγt (also seen on Th17 cells) [91]. IL-1β is also known to induce the expression of IL-6 from peripheral blood monocytes and epithelial cells [92, 93].

Whilst not only a driver of IL-17 production in AS, IL-1β and IL-1α are both known to play a role in normal fracture repair with both seen to be elevated in early and late phases of fracture repair [94]. The early elevation of IL-1 acts to activate osteoclasts independent of RANKL [95]. However, if levels are abnormally elevated in rat models, this creates a fracture site haematoma with thinner fibres and a denser clot structure, inhibiting migration of stem cell populations towards the fracture site [96]. Elevated levels of IL-1β and the associated changes in normal healing can lead to non-union fractures or inflammatory diseases resulting in abnormal bone resorption [97]. Of note is that deficiency of the IL-1 receptor antagonist in humans, termed (DIRA), is associated with new bone formation in the spine [98], indicating that unrestrained IL-1 family activity may lead to new bone formation.

IL-6 is a pleiotropic cytokine involved in a range of biological processes both inflammatory and anti-inflammatory [99]. That IL-6 is important in AS is argued against by failed trials of anti-IL-6R blockers in improving AS symptoms [100]. However, like IL-1, this cytokine is important in regulating the IL-23/17 axis where IL-6 is considered a key driver in the differentiation of de novo Th17 T cells in conjunction with IL-23 [101–103]. As mentioned above, the spinal enthesis harbours resident CD4+ and CD8+ T cell populations; both secrete IL-17A and TNF [23]. Importantly, IL-6R polymorphism has been linked to AS in large GWAS studies [104]. High levels of IL-6 are found in biopsies of sacroiliac joints of AS patients [105]. Circulating levels of IL-6 correlate with spinal inflammation [106, 107], with a significant reduction in IL-6 levels after TNF blockade [108]. In murine models, prolonged mastication caused accumulation of Th17 cells associated with subsequent gingival tissue repair that is driven by IL-6 upregulation in response to physiological tissue damage [109]. This ties into how both IL-17A and IL-6 affect the gastrointestinal tract beyond the oral mucosa where it where it has an important tissue protective role in intestinal barrier function [110, 111].

IL-6 is expressed by osteoblasts after a fracture has occurred due to the rapid increase in local IL-1β [94, 112]. The levels of IL-6 decrease over the course of the fracture repair [113], which is supported by the suspected role IL-6 plays in fracture healing. Where knockouts of IL-6 in mice fractures causes a significant reduction in osteoclastogenesis and impaired callus strength, though by 6 weeks, post-fracture most of the biomechanical features of the fracture were similar to wild type mice [114]. IL-6 also has a chemotactic effect upon resident mesenchymal cells, which migrate towards the site of the fracture [94].

However, abnormal and prolonged upregulation of IL-6 can cause significant bone degradation [115]. In murine models, elevated levels of IL-6 among other cytokines discussed causes an upregulation in STAT3 activation in osteoblasts and local fibroblasts [116]. This activation of STAT3 was seen to promote expression of RANKL and subsequent osteoclastogenesis, creating a positive feedback loop resulting in the prolonged inflammation and joint destruction associated with RA [116]. Blockading of IL-6 signalling is seen to ameliorate osteoclastogenesis and joint destruction in both animal models [117, 118]. With IL-6 signalling through JAKs [119], inhibition of JAK is becoming a more viable target for SpA therapies due its indirect and direct signalling through other cytokines mentioned above [120].

Another cytokine of interest in SpA is IL-22 since it is downstream of IL-23 and induced by IL-23 signalling. Its receptor IL-22Rα is not expressed on immune cells which creates a uni-directional signalling pathway, meaning only immune cells are seen to express IL-22, with CD4+ T cells [121], CD8+ T cells [122, 123], γδ T cells [124] and natural killer T cells [125] all being capable of IL-22 expression. However, in ILC populations, RORγt activation is required to induce IL-22 expression; this is due to the induced IL-23R upregulation which is the IL-23 receptor that induces IL-22 expression form immune cells [126]. The regulation of IL-22 expression by IL-23 is seen when mTOR is blocked in neutrophils there is subsequently inhibited IL-22 expression [127]. The IL-23 signalling axis is also shown to drive activation of T cells, which are well known to secrete pro-inflammatory cytokines associated with AS such as IL-22 and IL-17A [18, 128]. The use of IL-23 inhibitors in AS patients for a phase two trial showed no meaningful clinical improvements [13], with rat SpA models showing the same results, unless anti IL-23 is used prophylactically when there was suppression of IL-22 [87]. This suggests at IL-22 production in a potentially independent manner from IL-23, where IL-23 is needed for disease initiation but not its continuation or progression.

IL-23, which regulates IL-22 expression, is upregulated following femoral fracture in mice and likely explains upregulation of IL-22 expression [129]. IL-22 drives the migration of MSCs towards a site of injury where elevated levels of IL-22 could be present [29]. Osteogenic differentiation from MSC is enhanced by elevated IL-22; subsequently, IL-22 activates osteoblast induced bone remodelling via phosphorylation of STAT3 [18, 29]. Interestingly, mouse primary osteoblasts display only low levels of the IL-22Rα receptor, though its expression was upregulated when primed with bone morphogenetic protein (BMP)-2 [130].

## Emerging cytokines in enthesis fine tuning—IL-4 and IL-13

There has recently been a revaluation into the role of the traditional Th2 cytokines and the SpA field. IL-4 and IL-13 are classical Th2 cytokines, with a documented role in atopic dermatitis and asthma [131]. As well as driving Th2 differentiation, these cytokines are involved in IgE class switching. The many shared functions of IL-4 and IL-13 is related to signalling through the common shared receptor (IL-14Rα). Recent data in atopic dermatitis patients undergoing dupilumab treatment (IL-4 and IL-13 blocker) reported the development of peripheral enthesitis [132]. These observations have also been supported by other case reports of enthesitis development following dupilumab therapy [133].

The mechanism surrounding IL-4/IL-13 blocking-induced enthesitis are presently unknown, but genetic associations of unknown functional significance between IL-13 and psoriatic arthritis have been reported [134–136]. It is also of note that the IL-13 genetic associations do not appear to occur in AS, given the many subtle differences between these diseases. It could be hypothesised that IL-4 and IL-13 blockage could change the differentiation of immature T cells from a Th2 phenotype to a Th1/Th17 phenotype with subsequent increased IL-17/IL-22 and TNF production. In support of this, we recently showed that IL-4/IL-13R cells are present in the normal enthesis, and the synovial fluid of PsA patients contains measurable IL-4 and IL-13 [137]. IL-4+ T cells are also present at the normal enthesis and IL-4/13 reduced Th17 cytokines from stimulated entheseal T cells [137]. It has been known for over 20 years that IL-4 has a protective role in cartilage destruction, including that induced by IL-17 [138]. In the murine CIA arthritis model, both IL-4 and IL-13 reduced symptoms and bone erosion [139, 140]; however, this model at a pathological level is far closer to RA than SpA. The role of IL-4 and IL-13 in bone modelling has also been studied with KO models. IL-4 or IL-13 KO mice both present with reduced cortical bone mass [141]. In a murine fracture model, net bone formation or mineral deposition was not effected by either IL-4 or IL-13 KO; however, subtle perturbations in associated vascularisation and innervation were noted [142].

## Degenerative changes at spinal and peripheral enthesis and tissue repair

Normal spine and peripheral entheses are subject to age-related degenerative changes around the annulus and other entheseal structures. Even in AS, there is ample evidence of substantial degenerative changes reported around the spinal discs [143], so theoretically, such changes may be important in what is considered as mainly inflammatory disease. There is very little specific research relating to degenerative enthesopathy in the spine but more data from the peripheral skeleton including tendinopathy including the Achilles tendon. This latter process is not focussed at the insertional site but more proximal at the synovio-entheseal complex structure [144].

**Fig. 3:**
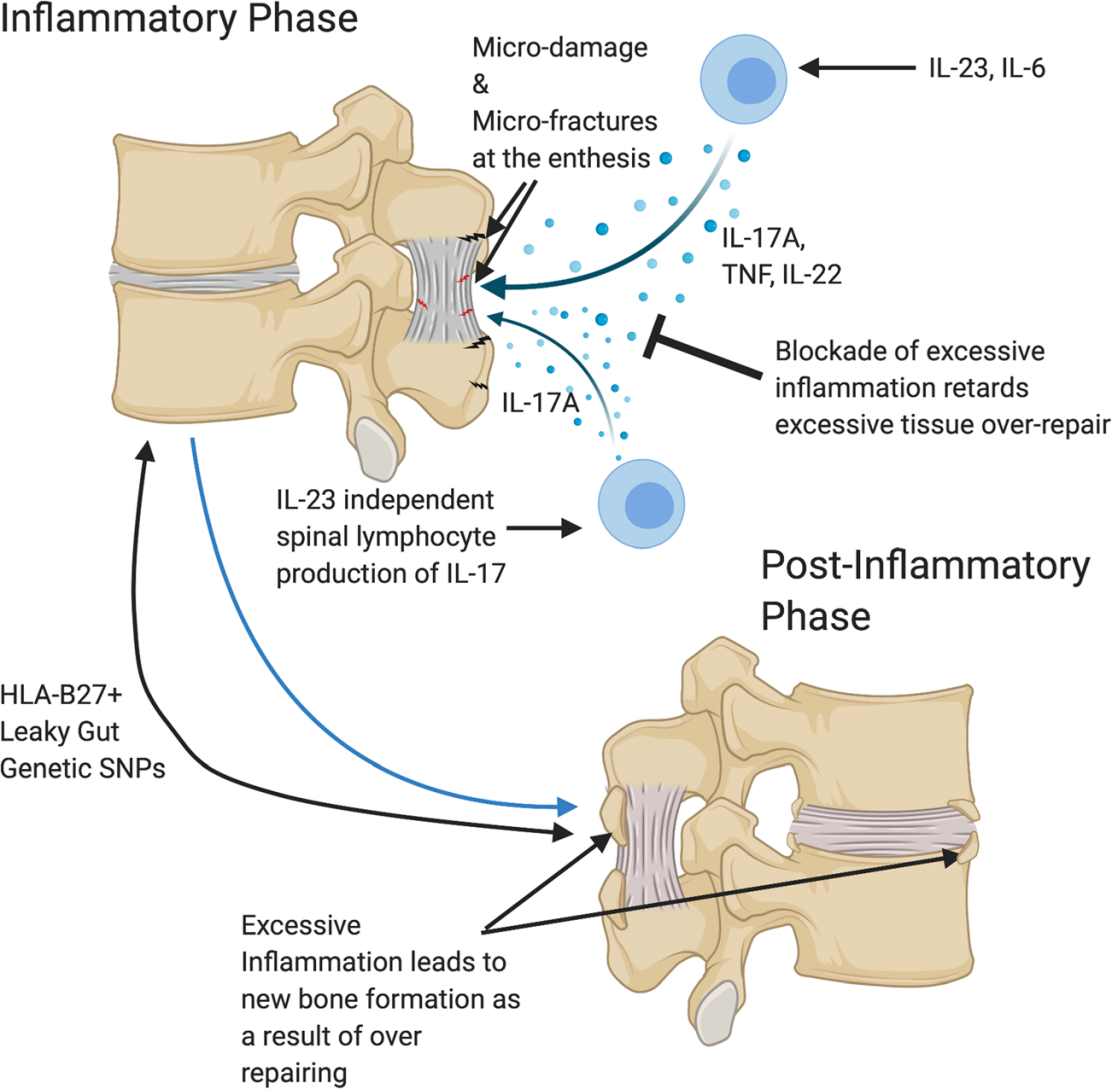
Overactivation of the immune system in tissue damage and excessive bone repair responses in ankylosing spondylitis. SNPs in PGE2 and IL-23/17 axis cytokines and others are clearly linked to spinal inflammation and post-inflammation repair. It is proposed that the initial microdamage to the spinal enthesis as shown in Fig. 1 leads to inflammation an immune driven tissue repair response. The same cytokine TNF and IL-23/17 pathways are responsible for both inflammation and also contributing to excessive repair responses. The dysregulation of homeostatic fine tuning of tissue repair, thus results in the characteristic post inflammation disease phenotype

**Fig. 4:**
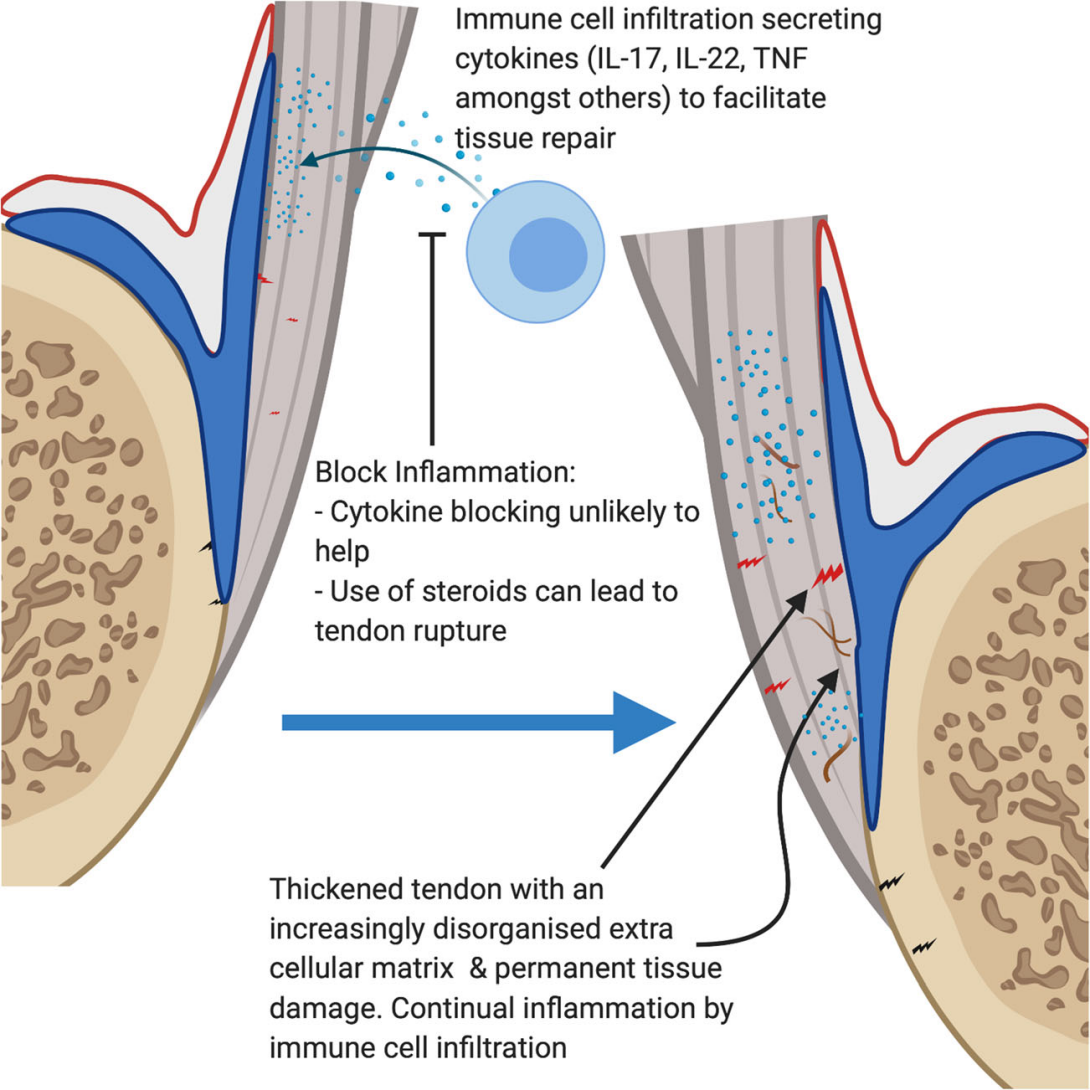
Inflammation in tendinopathy. The synovio-entheseal complex with tendons wrapping around bony tuberosities shows how the enthesis is more than merely a focal attachment site leading to stress dissipation over a wide area. Micro-damage at the Achilles tendon enthesis organ in chronic tendinopathy at this and other tendon locations near attachment points leads to immune cell infiltration to aid in tissue repair. However, unlike SpA, the excessive microdamage and injury may lead to persistent and elevated local immune responses or increased immune system fine tuning in an attempt to restore homeostasis. Prolonged inflammation may in theory contribute to tendinopathies, with thickening of the tendon and gradual loss of extra cellular matrix organization weakening the tendon. Unlike SpA where cytokine blockade efficiently controls disease by modulating the excessive immune fine tuning of cytokine homeostasis in a normal biomechanical environment, the situation here is different. The primary issue may be an abnormal biomechanical environment where the cell infiltration and associated physiological increase in fine tuning represents a repair attempt. The use of corticosteroids can lead to tendon ruptures highlighting an important role in a controlled amount of inflammation in tissue repair

The early phases of degenerative tendinopathies have a clear signature of an inflammatory phenotype [145]. Shortly after damage, rapid accumulation of neutrophils is seen in tendons, with subsequent macrophage accumulation over time [145], though more immunologically active cells are also seen to be elevated in early pathogenic tendons, namely populations of T cells, natural killer cells and mast cells [146, 147]. Significant elevations of IL-17 are seen in samples of early tendinopathy taken from the subscapularis, compared to both torn supraspinatus and healthy control tendons [147]. Tenocytes in culture when stimulated by IL-17 significantly increase secretions of TNF, IL-6 and IL-8 which is supported by the gene analysis of human tendinopathy patients [147, 148]. Not only is there an increase in cytokines with IL-17 stimulation, with resultant tenocytes collagen type III deposition as opposed to the healthy collagen type I phenotype, which creates a weaker overall tissue structure due to the loss of organization [147, 149]. Accordingly, immune activation in site of excessive damage may represent an exuberant attempted repair response rather than a detrimental one that is seen in axial SpA (Fig. 3). The detrimental impact of corticosteroids on tendinopathy may in part be due to antagonism of this immune-driven repair element [15, 150] (Fig. 4). Just like corticosteroids, it may be that the TNF and IL-17 will not impact on tissue repair or provide benefit in these more degenerative settings. In the spine, there is no compelling evidence that TNF blockers impact beneficially on degenerative disease nor do they exacerbate [151]. This supports the cytokine fine tuning theory of entheses and sites of high stress. Dysregulation of this fine tuning in immune driven inflammation can be usefully antagonized with TNF and IL-17 pathway inhibition, but we expect that ongoing efforts to antagonize attempted cytokine fine tuning towards repair in primary degenerative or biomechanically related enthesopathies will fail.

## Conclusions

It is clear that the normal enthesis has a resident immune system, and in experimental studies, there is ample evidence that this plays a role in the normal entheseal tissue repair. Excessive tissue repair is the hallmark of human spinal enthesitis-related pathology, and certain cytokines including TNF and IL-17A are clearly involved as demonstrated by human studies. Akin to the skin and the gut, dysregulation of the enthesis resident immune system can often lead to disease states affiliated with initial destruction but eventual excessive tissue repair. The exact pathogenesis of these disease states is complicated due to the built-in redundancy seen within the immune system. In more degenerative-associated enthesopathy states, the attempted cytokine and immune fine tuning may be part of a commensurate and attempted repair response, the suppression of which may have no benefit or be detrimental.
